# Facial emotion recognition using deep quantum and advanced transfer learning mechanism

**DOI:** 10.3389/fncom.2024.1435956

**Published:** 2024-10-30

**Authors:** Shtwai Alsubai, Abdullah Alqahtani, Abed Alanazi, Mohemmed Sha, Abdu Gumaei

**Affiliations:** ^1^Department of Computer Science, College of Computer Engineering and Sciences, Prince Sattam Bin Abdulaziz University, Al-Kharj, Saudi Arabia; ^2^Department of Software Engineering, College of Computer Engineering and Sciences, Prince Sattam Bin Abdulaziz University, Al-Kharj, Saudi Arabia

**Keywords:** facial expressions, artificial intelligence, deep learning, quantum computing, ResNet model

## Abstract

**Introduction:**

Facial expressions have become a common way for interaction among humans. People cannot comprehend and predict the emotions or expressions of individuals through simple vision. Thus, in psychology, detecting facial expressions or emotion analysis demands an assessment and evaluation of decisions for identifying the emotions of a person or any group during communication. With the recent evolution of technology, AI (Artificial Intelligence) has gained significant usage, wherein DL (Deep Learning) based algorithms are employed for detecting facial expressions.

**Methods:**

The study proposes a system design that detects facial expressions by extracting relevant features using a Modified ResNet model. The proposed system stacks building-blocks with residual connections and employs an advanced extraction method with quantum computing, which significantly reduces computation time compared to conventional methods. The backbone stem utilizes a quantum convolutional layer comprised of several parameterized quantum-filters. Additionally, the research integrates residual connections in the ResNet-18 model with the Modified up Sampled Bottle Neck Process (MuS-BNP), retaining computational efficacy while benefiting from residual connections.

**Results:**

The proposed model demonstrates superior performance by overcoming the issue of maximum similarity within varied facial expressions. The system’s ability to accurately detect and differentiate between expressions is measured using performance metrics such as accuracy, F1-score, recall, and precision.

**Discussion:**

This performance analysis confirms the efficacy of the proposed system, highlighting the advantages of quantum computing in feature extraction and the integration of residual connections. The model achieves quantum superiority, providing faster and more accurate computations compared to existing methodologies. The results suggest that the proposed approach offers a promising solution for facial expression recognition tasks, significantly improving both speed and accuracy.

## 1 Introduction

Facial expressions are a form of non-verbal communication that arise from the movement of facial muscles to convey emotions or gestures (Khan, 2022). They serve as a means of expressing emotions, such as opinions, goals, intentions, and feelings. However, predicting human expression is challenging. Currently, computer applications are widely used to calculate facial expression scores. Facial emotion recognition (FER) is essential for computer vision-aided applications to enhance human–computer interactions.

Human faces exhibit a heterogeneous nature, with image variations caused by factors such as lighting and poses, which pose challenges for computer models to achieve robust and accurate predictions (Kaur and Singh, 2022). In FER, the process of associating different facial expressions with their corresponding emotions involves several steps, including image pre-processing, feature selection, and feature classification.

In traditional computer-based models, feature extraction and noise reduction have been carried out using polyp (Tsuneki, 2022) computer-aided classification models. Various feature extraction techniques have been used in existing research, such as principle component analysis (PCA) (Sachadev and Bhatnagar, 2022), linear discriminant analysis (LDA), individual component analysis (ICA), local dynamic pattern (LDP) (Makhija and Sharma, 2019), geometric feature mapping (Rosen et al., 2021), and elastic bunch graph mapping (EBGM) (Oloyede et al., 2020). Machine learning (ML)-based algorithms can be used in the classification process. However, an additional feature engineering process is required for feature extraction. Deep learning (DL) (Karnati et al., 2023), a sub-domain of ML algorithms, has been widely used in image classification tasks for enhanced accuracy. The training time for DL algorithms has been less than for ML algorithms. Convolutional Neural Network (CNN) (Mohan et al., 2020) is a significant algorithm used for image classification as part of ML and deep learning-based neural networks (Mungra et al., 2020; Mohan et al., 2021). Unlike the traditional models, CNN can extract abstract and accurate features. Automatic learning can be enhanced with CNN by adopting depth features (Karnati et al., 2022) and block architectures. Traditional CNN algorithms perform better for many image classification tasks like SVNN (Ghasemi et al., 2020), CIFAR (Yang et al., 2020), and MNIST (Kadam et al., 2020).

Quantum-based principles can be integrated into ML models across various domains. Quantum-enabled ML models have been used in various algorithms such as quantum neural networks, quantum generative models, and quantum support vector machines. Artificial intelligence (AI)-based algorithms can be seen as a resemblance of the human brain with highly abstract functions. Significant AI models include capsule neural networks (Jiang et al., 2020), recurrent neural networks (RNN) (Mei et al., 2019), feedforward neural networks, (Tacchino et al., 2020) and CNN. Quantum neural networks (QNN) employ quantum mechanisms to enhance the structure of neural networks (Wang et al., 2022). The architecture can be improved through the concepts of quantum interference attributes, quantum entanglement, and quantum parallelism. The performance of a traditional neural network can be enhanced by implementing a conventional neural network with a quantum neural network. The hybrid architectures thus formed can be trained and tested on IBM Quantum Experience through Qiskit-enabled quantum computers.

QNNs have similarities with traditional neural models and have variation parameters. QNNs have several potential advantages. Quantum computers can outperform traditional models in speed for Fourier transform based on Shor’s factoring technique. Various computational issues can be efficiently resolved with quantum contextuality and non-locality. Moreover, the learning process from a quantum dataset created by a quantum process is more efficient than a traditional dataset. In large-scale exponential datasets such as Hilbert space, the ability of QNN to extract adequate data from the quantum state is difficult (Li et al., 2022).

Moreover, quantum networks can perform massive parallel calculations and provide high-performance speed. An attention mechanism has recently been used in QNN models. An enhanced CNN model has been used in a DL computer vision application named AlexNet. It has performed data augmentation, convolutions, ReLU activations, max pooling, stochastic gradient descent (SGD) (Zheng et al., 2019), and dropout. The issue with deep network training can be mitigated by implementing modified blocks that ignore and leap over layers. This enhanced the training of large networks with fewer training errors.

Another ResNet model has been implemented for deep-coupled low-resolution neural networks (Kavitha et al., 2022). The ResNet model has selected dissimilar features in various facial images. The image features have been projected with training from coupled mappings of branch networks. The models have been evaluated with SCface datasets and LFW datasets and have achieved remarkable accuracy for face verification (Singhal et al., 2021). Even though various face recognition models have been developed, high recognition rates are difficult to achieve with traditional feature classification algorithms.

Moreover, convolutional layers have the ability to handle only spatial features in images. Subtle and depth features are not properly recognized with CNN models. Furthermore, the abstract features extracted in the deep CNN model suffer from vanishing gradient issues as the number of layers increases. QNN algorithms provide correlated and probabilistic components, whereas performance is limited by dimensionality issues and computational bottlenecks. To resolve all the above issues, the MuS-BNP with ResNet-18 model named MuS-BNP is proposed.

The model uses the FER 13 dataset to predict the facial emotions in the images. Unlike traditional CNN and ResNet architecture, both shallow and deep features are extracted using a backbone stem integrated with a quantum convolutional layer. This layer incorporates various parameterized quantum filters, which replace the conventional kernel in traditional convolutional layers. The parameterized quantum filter is used to obtain quantum bit information in the local data space. It includes a double-bit gate that performs quantum entanglement on other quantum bits, enhancing the interaction between data points.

In this process, pixel value information is converted into quantum state information through quantum state encoding, achieved via a quantum rotation gate. The model retains the weight-sharing mechanism of the traditional kernel while incorporating quantum parameters to boost computational capabilities. Furthermore, the filter connection phases in the ResNet-18 model are linked with the MuS-BNP through residual connections, which significantly enhance computational performance. The major contributions of the proposed model, combining the MuS-BNP with the ResNet-18 architecture, are as follows:

- To perform shallow and deep feature extraction through a backbone stem network and a modified quantum convolutional layer with parameterized quantum filters.
- To perform facial emotion classification through the proposed MuS-BNP with the ResNet-18 model in less computation time.
- To evaluate the efficacy of the proposed model with performance metrics such as accuracy, F1-score, recall, and precision.

### 1.1 Contributions

QNNs are typically designed to handle large data efficiently, unlike conventional NNs (neural networks), which permit them to accomplish better classification. The present study proposes ResNet18 architecture with a Modified Sampled Bottleneck Process for FER. Accordingly, residual connections have been utilized to associate the filter connection phase in the ResNet-18 model with the MuS-BNP. This architecture helps manage computational efficiency while leveraging the benefits of residual connections. Moreover, the residual version of the ResNet-18 model with the MuS-BNP has employed a simplified module.

Furthermore, the filter expansion layer that follows each module has been enlarged with the dimensions of the filter bank. For matching the input, it has been integrated before. Thus, it reimburses the minimization of dimensionality that is available in an n block.

Feature extraction has also been accomplished with the quantum convolutional layer. This is encompassed with various parameterized quantum filters. Similar to the convolution kernel present in the conventional convolutional layer, the parameterized quantum filter finds utility for information extraction that is present in individual quantum bits. In an image, the pixel value corresponding to the information has been altered into the quantum state information (that utilizes quantum state encoding) with the means of the quantum rotational gate R (ɵ). In accordance with this process, the procured information regarding the features of the image has been modified into the angle of the quantum rotatory gate.

Furthermore, for the quantum rotatory gate, the corresponding parameters have been afforded by each pixel value. The proposed method comprises exclusive quantum mechanical features and retains the weight sharing in the convolutional kernel. In the proposed technique, individual blocks have a self-regulating convolutional way of delivering information in the prior and middle layers.

The strategy introduces the concept of “pass-over,” a modification from the ResNet model that builds on modest blocks containing residual connections. The traditional residual building block has not utilized the information accessible in the middle layer. However, the proposed model incorporates pass-over information to capture all relevant features.

Thus, the proposed ResNets with QNNs possess the ability to generalize. Furthermore, by leveraging the effects of quantum-like superposition and entanglement, QNNs obtain several complex associations amongst the input features, resulting in model robustness and better generalization. The proposed QNN could effectively use quantum hardware, leading to the count of quantum gates needed for computation. Through this system, quantum gates needed for computation are also minimized. The proposed framework finds more complex and subtle features of an image than traditional algorithms, resulting in robust and optimal classification. Moreover, the proposed system performs functions on multiple qubits at concurrent times, permitting the effective parallel processing of the features from the images.

### 1.2 Paper organization

Section II of the paper deals with the review of existing literature for image recognition and classification through various ML models, DL models, and quantum-based DL models. The problems identified from the existing literature have also been discussed. Section III deals with the proposed flow, architecture, and mathematical formulations. Section IV deals with the dataset description, performance results, comparative results, and discussions. Section V deals with the conclusions and future recommendations of the work.

## 2 Review of literature

Image classification and emotion recognition can be performed in literature through various ML algorithms, DL algorithms, and enhanced quantum-based ML and DL algorithms. The section briefly deals with all conventional models, along with the gaps identified from the state of artworks.

A human emotion identification model has been proposed in the study (Alreshidi and Ullah, 2020) using two ML algorithms for image classification and detection. The model has been trained for real-time implementations offline. The faces in the image are initially recognized with AdaBoost cascade algorithms (Chen et al., 2019). The facial features denoted by localized appearance data named Neighborhood Difference Features (NDF) (Kaplan et al., 2020) have been extracted. The association among various NDF patterns has been considered rather than intensity data. Even though the study calculates only seven facial emotions, it can be extended to more facial feature recognition. The model has been invariant to skin color, gender, orientation, and illumination. The evaluation results on Real-World Affective Faces (RAF) (Jiang et al., 2020) and Static Facial Expressions in the Wild (SFEW) (Liu, 2020) datasets have exhibited 24 and 13% accuracy enhancement, respectively.

Another study has been designed to identify microexpressions in human faces. Unsupervised micro-expression detection models based on ML algorithms have been suggested with extreme learning machines (ELMs). The algorithm offers higher performance and faster training ability than conventional algorithms. The ELM model has been compared with the Support Vector Machine (SVM) (Okwuashi and Ndehedehe, 2020) benchmark model for training time efficacy. Feature extraction has been performed through Local Binary Pattern (LBP) (Zhao et al., 2019) on apex-micro expression frame and Local Binary Pattern on Three Orthogonal Planes (LBP-TOP)-based division of image segments from video through spatiotemporal features. The model has been evaluated using a dataset from the Chinese Academy of Sciences (CASME II). The results indicate that ELM has a better prediction rate and less computation time than SVM (Adegun and Vadapalli, 2020).

The facial emotion intensity has been encoded by considering multimodal facial behavior for recognizing emotions from intensities. The intensity extraction has been performed with ML algorithms like Random Forest (RF) (Speiser et al., 2019), SVM, and K-Nearest Neighbor (KNN) (Ma et al., 2020). Three feature extraction methods, namely local binary pattern (LBP), histogram of oriented gradients (HOG) (Zhou et al., 2020), and Gabor features (Munawar et al., 2021), have been implemented. Intensity calculation and emotion identification have been performed through a comparative analysis of three algorithms on CK, B DFE, JAFEE, and private datasets. Emotion recognition and facial intensity detection have been analyzed from the three algorithms (Mehta et al., 2019).

Another fake image detection model has been developed with generative adversarial networks (GANs) that create fake images with low-dimension noise. Fake images have created various issues in social media networks. Contrastive loss-based fake image detection has been implemented using the DL-based DenseNet model. Pairwise information has been fed as input through a two-streamed network model. The training has been performed on the pairwise information to identify the fake input image (Hsu et al., 2020). DL-based CNN models have exhibited high computational efficiency and unsupervised feature extraction. CNN-based image prediction has been performed on the FER 2013 dataset. The visual geometric group (VGG) algorithm (Deepan and Sudha, 2020) has been used to design the model with various learning schedulers and optimization techniques. The model’s hyperparameters have been tuned, and the accuracy is 73.28% (Khaireddin and Chen, 2021).

High-level feature identification from facial images has been performed with a two-layer CNN model and sparse representation. The training data independent of feature space has been used to sparsely denote the facial features in the proposed Sparse Representation Classifier (SRC). Real-world classification and feature recognition depend on the proper details extracted from the faces of images. The results of the SRC-based feature selector have proved superior to other traditional classifiers (Cheng et al., 2019). The transfer learning (TL)-based deep CNN (DCNN) model has been developed for accurate classification of images, considering shallow and depth features. The pertained DCNN model has been modified with a FER-compatible upper dense layer fine-tuned to recognize facial emotion. The pipelining technique has been adopted after dense layer training and tuning. The model has been tested on pertained DCNN models like DenseNet-161 (Song et al., 2019), Inception-v3, ResNet-152 (Gour et al., 2020), ResNet-50, ResNet-34, ResNet-18, VGG19 and VGG-16, along with JAFFE and KDEF, using a 10-fold cross-validation approach (Akhand et al., 2021).

Another study identified facial emotion from video sequences with global and local networks (Hu et al., 2019). The cascaded CNN-LSTM networks and Local Enhanced Motion History Image (LEMHI) (Gavade et al., 2022) have been implemented for the above feature extraction. LEMHI has been used to aggregate the video frames as a single frame, which has been fed into the CNN for prediction. The global features have been extracted through an enhanced CNN-LSTM model as a classifier and feature extractor. The final prediction was performed using a late fusion fashion-based random search summation model. The information to decode the features from facial images has been obtained from each CNN layer. The experiments on MMI, CK+, and AFEW datasets have exhibited better integrated model performance than the individual model. The complexity of the CNN (Jing et al., 2022) model depends on the activation function.

Although the ReLU activation function outperforms tanh and sigmoid in many cases, it still has limitations. The ReLu model returns zero value on negative inputs, which is termed neuronal necrosis. This has been eliminated by implementing a piecewise activation function in CNN. The new function has been compared with other functions such as softplus-ReLu, leaky ReLu, tanh, and Sigmoid (Zhang et al., 2022). The comparison of results on the Keras framework utilizing the FER13 and JAFFE datasets exhibited better activation function performance (Wang et al., 2020). Another deep CNN-based model has been implemented with residual blocks for enhanced performance. The image labels have been initiated, followed by training on the proposed DNN model. Japanese Female Facial Expression (JAFFE) and Extended Cohn–Kanade (CK+) datasets have been used to test the accuracy of the model (Jain et al., 2019). Computational issues have been optimized through an unsupervised ensemble model of hybrid deep neural networks (HDNN) and an improved quantum-inspired gravitational search algorithm (IQI-GSA). Quantum computing and gravitational search algorithm (GSA) have been combined to form IQI-GSA. The local trapping and stochastic features have been handled with the enhanced model. The temporal and relational components have been optimized by hybridizing recurrent and convolutional (HDCR-NN) neural models. The experimental analysis has been performed on KDEF and JAFFE datasets to exhibit the model’s efficacy (Kumar et al., 2021).

Transfer learning (Tammina, 2019) with a quantum-based hybrid approach has been implemented to ensure security and reliability. The fake images have been classified using the ResNet-18-based quantum neural model. The model has been trained on various depths, and the reliability of vision-based models is tested (Ciylan and Ciylan, 2021; Kumar et al., 2022). The kernel-based quantum CNN model has been implemented to diagnose pneumonia early. The hybrid model can detect pneumonia from chest X-ray images obtained from a public repository. High classification accuracy has been obtained with the inclusion of a quantum model (Tayba et al., 2022). A parameterized circuit-based quantum deep convolutional neural network (QDCNN) model has been proposed in another study to classify image emotions. Quantum-classical training has been implemented through variational quantum algorithms. Parameters have been updated through QDCNN, and complexity has been analyzed using GTSRB and MNIST datasets to evaluate validity and feasibility (Li et al., 2020).

Tensorflow quantum-based (Lazzarin et al., 2022) QCNN models have been implemented for binary image classification. Box-counting-based fractal features, multi-scale entanglement, and the renormalization ansatz model have been used for downscaling, followed by classification through hybrid QCNN on the breast cancer dataset (Chen et al., 2022). Particle swarm optimization with binary encoding (BQPSO) based on quantum principles has been adopted to perform binary encoding of image emotions. A CNN model has been used to classify the features extracted from the hybrid model. The efficacy has been tested with seven benchmark datasets (Li et al., 2019). A quantum Hopfield network has been designed by combining quantum principles with traditional neural networks. The model has been applied to image recognition in a conventional computer, and its feasibility has been validated (Liu et al., 2020). Quantum Neural Networks (QNNs) have been evaluated for negational summary and binary classification in another algorithm on Google’s quantum computing platform (Dong et al., 2022).

Moreover, the FER is considered critical for several implementations. However, existing studies have shown better results in facial recognition. Moreover, the FER systems have shown enhanced accuracy in ML and DL methods compared to the conventional FER methods (Borgalli and Surve, 2022).

### 2.1 Problem identification

Various problems identified from the extensive literature have been discussed as follows:

- ML algorithms for facial expression recognition suffer from dynamic head motion, illumination variants, and noise sensitivity. Moreover, spatial and temporal features have not been integrated in the study. Furthermore, the work has not considered facial deformation and geometric features (Alreshidi and Ullah, 2020).
- Deep CNN-based models can handle spatial features alone in the FER 13 dataset (Jain et al., 2019). The vanishing gradient problem has occurred with an increase in the number of CNN layers. Training CNN-based models such as VGG, ResNet, and Inception requires significant computational power and large datasets (Akhand et al., 2021).
- Feature extraction capability in conventional shallow CNN models has been limited in the case of high-resolution images (Li et al., 2019).

## 3 Proposed methodology

**Figure 1:**
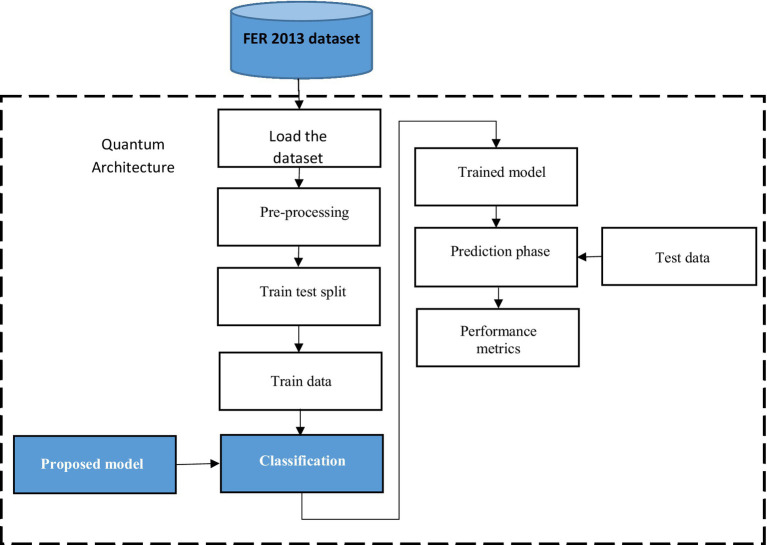
Classification of quantum image on facial expression with modified ResNet architecture.

The proposed study aimed to recognize facial expressions by employing quantum computing alongside the ResNet-18 model and the MuS-BNP architecture. However, many existing studies have intended to perform facial expression recognition. The accuracy of the already existing study is less and needs further improvement. In the present study, the information present in the qubits has been manipulated so that it is capable of producing more quality solutions to complex problems quickly. Hence, it is clear that quantum computing has been used to address difficult problems. The classification of quantum images based on facial expressions using modified ResNet architecture is shown in Figure 1.

The FER 13 dataset has been loaded and preprocessed. The process of preprocessing transformed the raw data into a usable format. The transformed data were then split into training and testing sets. A train test split has been used for the model validation procedure, which stimulates the model’s performance for new and unseen data, and the outcome of the train test split is trained data. The trained data was classified using the proposed ResNet-18 model with the MuS-BNP, which produces the trained model. Both the trained model and test data were used to predict the result. Performance metrics such as precision, recall, F-measure, and accuracy were used to assess the proposed model.

### 3.1 Quantum architecture

**Figure 2:**
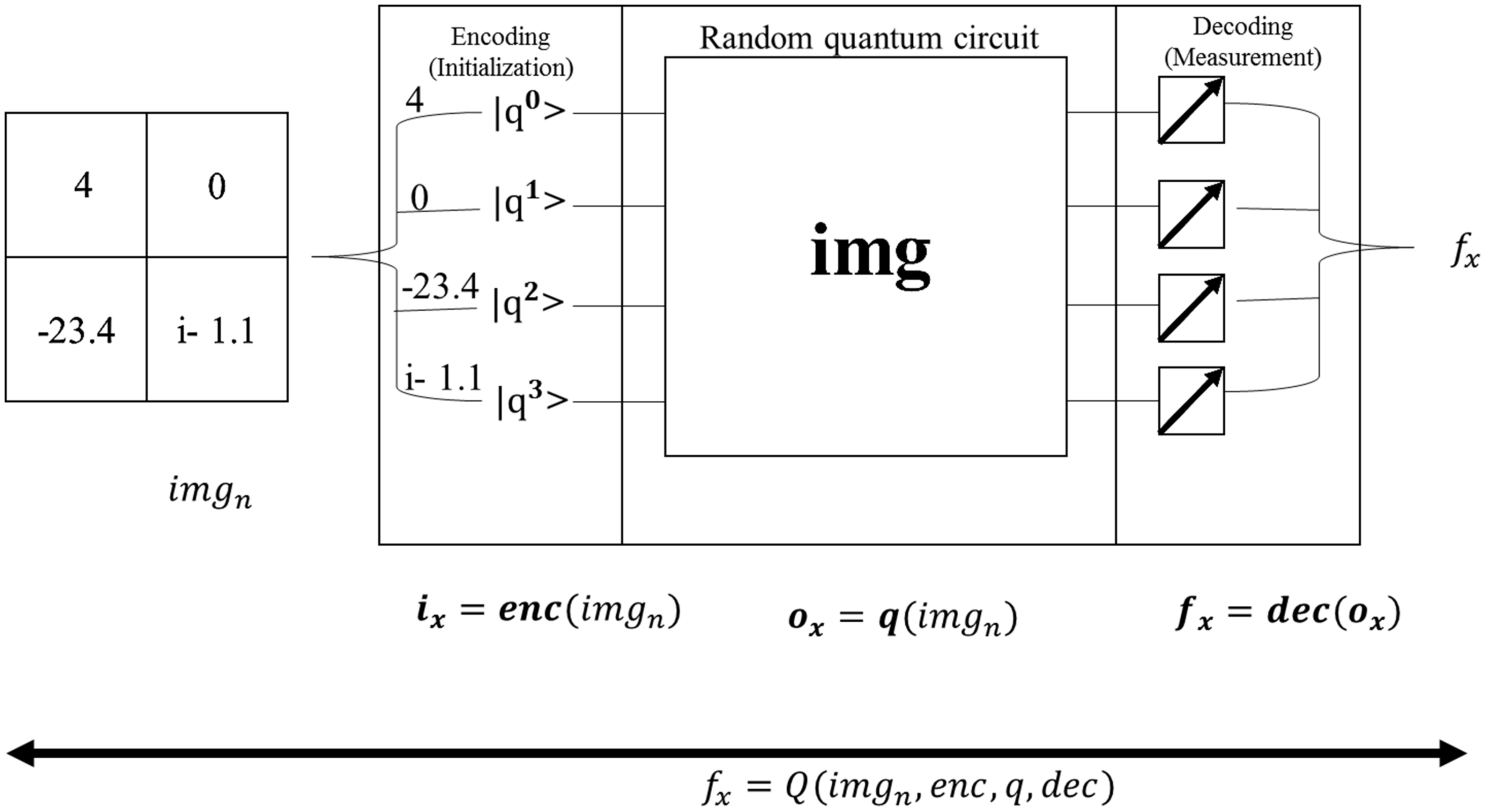
An in-depth look at the processing of classical data into and out of the random quantum circuit in the quantum convolutional filter.

When QCF is exercised on an input tensor, a feature map is produced by each QCF due to the spatial transformation of local subsections present in the input tensor using QCF. However, in contrast to the modest element-wise matrix multiplication that traditional convolutional filters have applied, QCF has used a quantum circuit to transform structured and random input data. In the present study, a quantum circuit, which is randomly generated, has been used in QCF, which is different from the designed structure. By using QCF, the process can be formalized and transforms the classical data as mentioned below:

1. Single QCF, which used random quantum circuit ‘ q ’ and a local subsection of images, has been taken as input from the dataset u . Each input has been defined as $u_{x}$ , and the matrix size of each $u_{x}$ is nbyn,wheren>1.
2. Though many ways are available to encode $u_{x}$ at the initial state of q , for each QCF, one specific encoding function e has been chosen, the encoded initialization state $ix\;as\;i_{x}=enc\left(img_{n}\right)$ has been defined.
3. After applying the quantum circuit to the initialized state ix , an output quantum state ox has been attained, which is the result of quantum computation where the relationship between ix and ox is given as $o_{x}=q\left(i_{x}\right)=q\left(enc\left(img_{n}\right)\right)$ .
4. Though many ways are available with a finite number of measurements to decode the information of ox, to confirm the consistency of QCF output with other similar output taken from regular classical convolution, the final decoded state has been given as $f_{x}=dec\left(o_{x}\right)=dec\left(q\left(enc\left(img_{n}\right)\right)\right)$ where d refers to the decoding function, and $f_{x}$ refers to a scalar value.
5. The complete transformation of $dec\left(q\left(enc\left(img_{n}\right)\right)\right)$ has been defined as QCF transformation at this point, in which Q of $u_{x}$ , aka $f_{x}=Q\left(img_{n},enc,q,dec\right)$ . A single QCF visualization has been shown in Figure 2, which exhibits the process of encoding, applied circuits, and decoding.
6. The number of classifications that happened when the classical convolutional filter was applied as an input from dataset u , the required number of computations is given as $O\left(n2\right)$ , placing the computational complexity squarely in P . It is not considered in the case of computational complexity Q . It has emerged from the complexity of random quantum circuit transformation q , where e and d show efficient performance on classical devices. Figure 2 illustrates the step-by-step QCF procedure in detail.

The present study has highlighted the novelties obtained from the QNN algorithm: the quantum convolutional layer generalizability inside a usual CNN architecture, the quantum algorithm’s ability to be used on practical datasets, and the efficient use of features presented by quantum convolution transformation. Later, research was conducted in the field of using quantum circuits in ML applications, in which randomly parameterized quantum circuits were used to process classical data and linear models were trained using the output. Quantum transformations have built the model and shown more benefits in comparison with further linear models, which are directly built on the dataset itself, but the level of performance is not the same when compared with other classical models. The experiments in the present study have been built on these results, in which quantum feature detection has been integrated into more difficult neural network architecture since the QNN framework introduced classical models that contain non-linearities.

### 3.2 ResNet18 architecture with modified up-sampled bottleneck process

A residual network employs residual blocks, which allow additive interaction between the input and output present in the two convolutional layers. The advantage of ResNet is given as a gradient that flows directly on identity function from future layers to past layers, which has partially solved the disappearing gradient problem. To improve the flow of information between the layers, original blocks replace the cascade blocks. Two Conv-BatchNorm-ReLU layers are used to build every cascade block, two in-out lines, and a shortcut connection line. However, the deep layer network contains many feature map inputs. To increase computational efficiency, the cascade block has been modified into a cascade bottleneck block, which uses four four-layer stacks instead of two.

In the present research, residual connections have been used to link the filter connection stage in the ResNet-18 model with the MuS-BNP. Therefore, the architecture allows for the maintenance of computational efficiency, which attains the advantages of the residual connection process. A residual version of the ResNet-18 model with the MuS-BNP has used a more simplified module. The filter expansion layer follows each module in which the dimensions of the filter bank have been enlarged. To match the input, it has been added before. Hence, it reimburses the reduction of dimensionality available in the n block.

**Figure 3:**
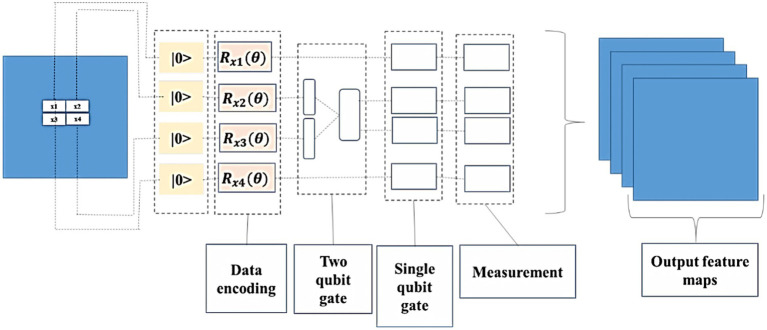
Quantum convolution layer.

Feature extraction was done through the quantum convolutional layer, which is composed of several parameterized quantum filters. Like the convolution kernel present in the traditional convolutional layer, the parameterized quantum filter has been used to extract the information present in every quantum bit, which exists in the data local space. A quantum filter consists of a double-bit gate in which quantum bit unitary conversion can be performed, and a double-bit gate is enforced on neighboring quantum bits, which leads to quantum entanglement present in neighboring quantum bits. In the image, the pixel value of the information has been changed into quantum state information (which uses quantum state encoding) using quantum rotation gate R(ɵ). Based on the process, the information attained about the features of the image has been altered to the angle of the quantum rotatory gate. Each pixel value has provided the corresponding parameters for the quantum rotatory gate. The quantum bit initial state |0 > has been acted by different quantum rotatory gates, and the quantum state stores the feature information. It can be utilized as model input to QNN. For instance, by considering n∗n , initially, the function of quantum feature extraction is encoded into the quantum state by coding the quantum bit. Furthermore, the quantum state has evolved by using a parameterized quantum circuit and, finally, by using expected value measurement outputs a real number. The method possesses both exclusive quantum mechanics properties and retains the sharing of weights in the convolutional kernel. Figure 3 shows the quantum convolutional layer.

**Figure 4:**
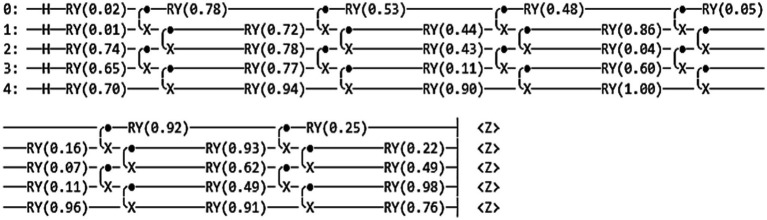
Proposed quantum circuit.

The present study has introduced the quantum circuit with parameters to enhance the network’s performance. Quantum filters include a rotary gate *R_y_*ɵ and a CNOT gate. Figure 4 shows the quantum circuit diagram.

ResNet has been used in computer vision applications as a DL model. Many convolutional layers have been supported by CNN architecture. ResNet-18 is a CNN that consists of 18 layers deep. The vanishing of the gradient has been improved by using the network. The improved algorithm has used ResNet-18. The existing study has optimized the input present in the network. The input features were extracted in parallel, and feature fusion was performed at the termination of the parallel structure. A specific method has been used to accept the three parallel routes. In the convolutional operations present in the multi-feature fusion, to confirm the integrity of the input image size, the step has been set to 1.

**Figure 5:**
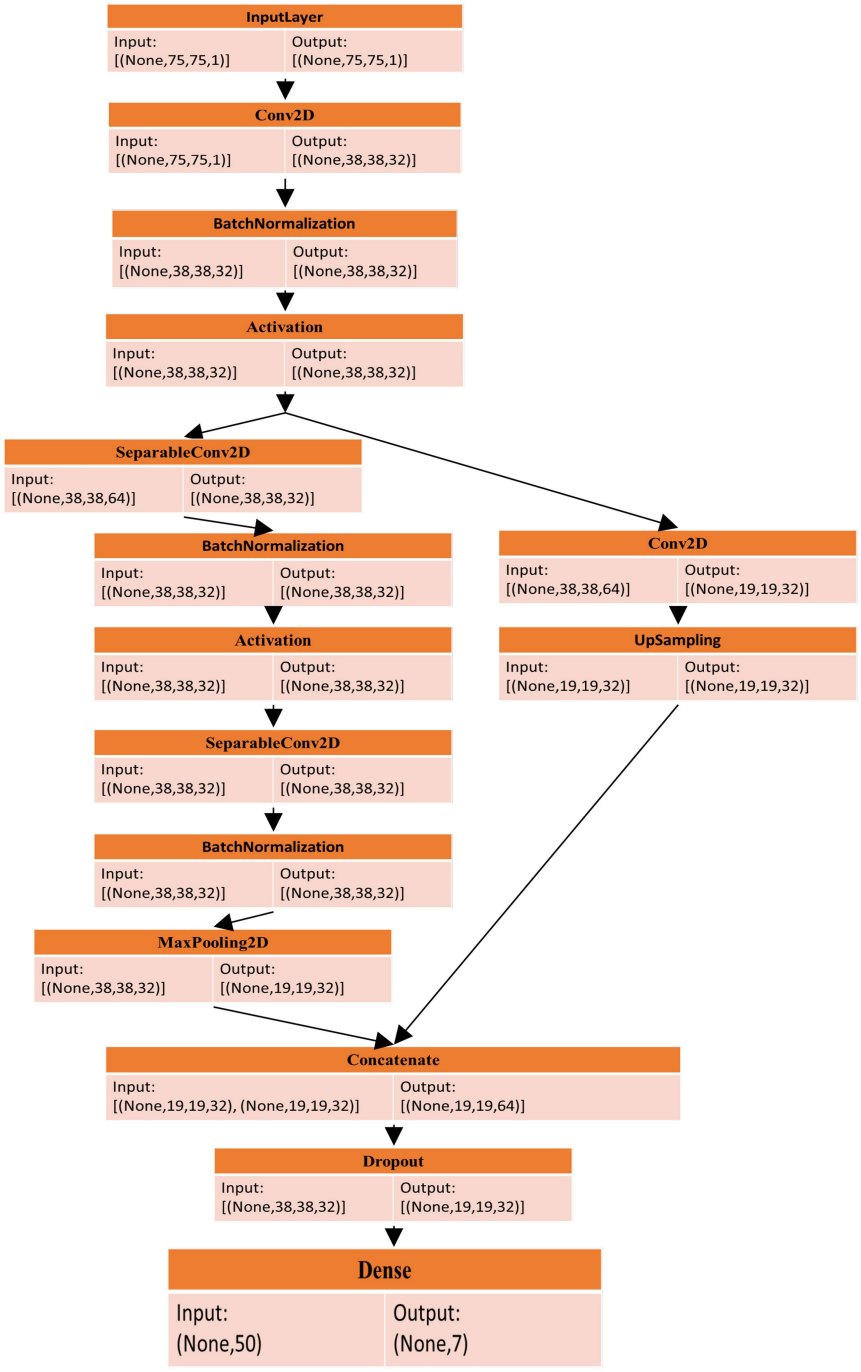
The flow of the ResNet-18 model with the MuS-BNP.

**Figure 6:**
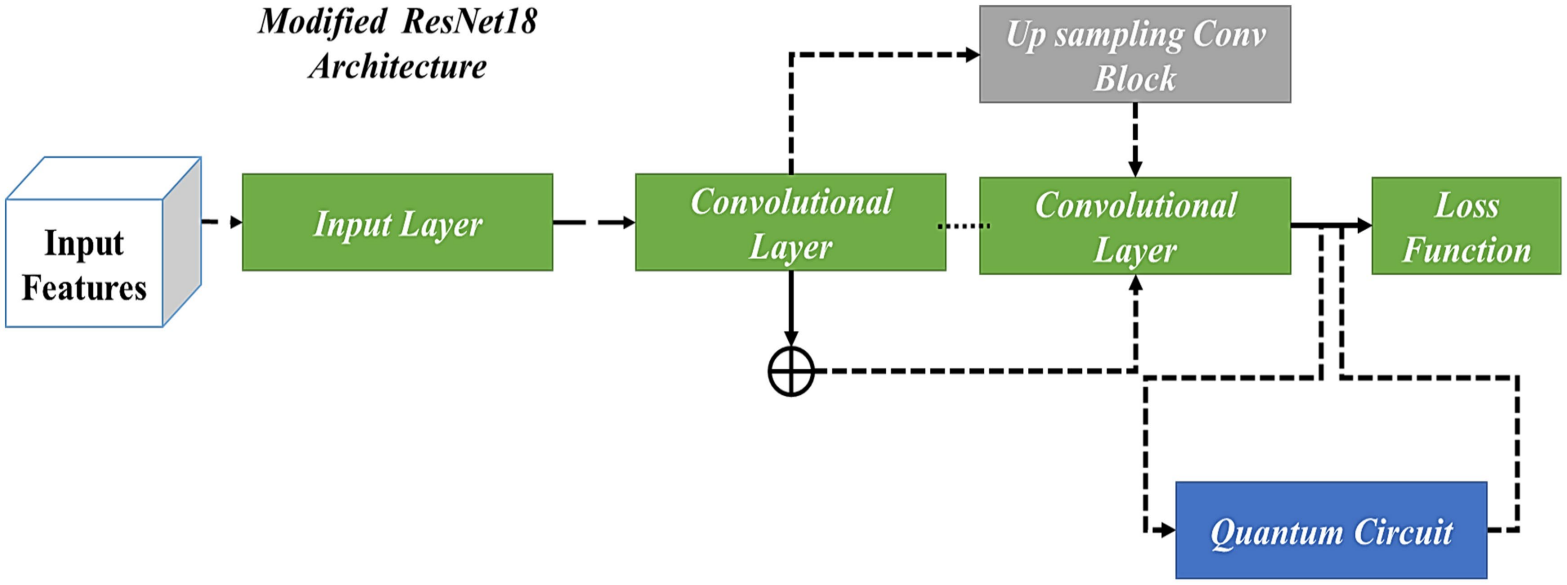
ResNet18 Architecture with Modified Up Sampled Bottleneck Process.

Figure 5 has been used to better understand the process. Similarly, when applying the initial residual unit, the number of feature layers is increased, and a better interpretation of dimensionalities is presented. In the end, the outcomes of three parallel routes were used for feature fusion, which extracts the features of the image and, in turn, improves the performance of the proposed model. The proposed QNN efficiently utilizes quantum hardware and reduces the number of quantum gates needed for a particular calculation. Moreover, the model outperforms traditional algorithms in identifying complex image features, improving classification accuracy and reliability. It also performs tasks on several qubits simultaneously, allowing for efficient parallel processing of image feature datasets. Figure 6 illustrates the modified up-sampled bottleneck process with the ResNet-18 architecture.

**Figure 7:**
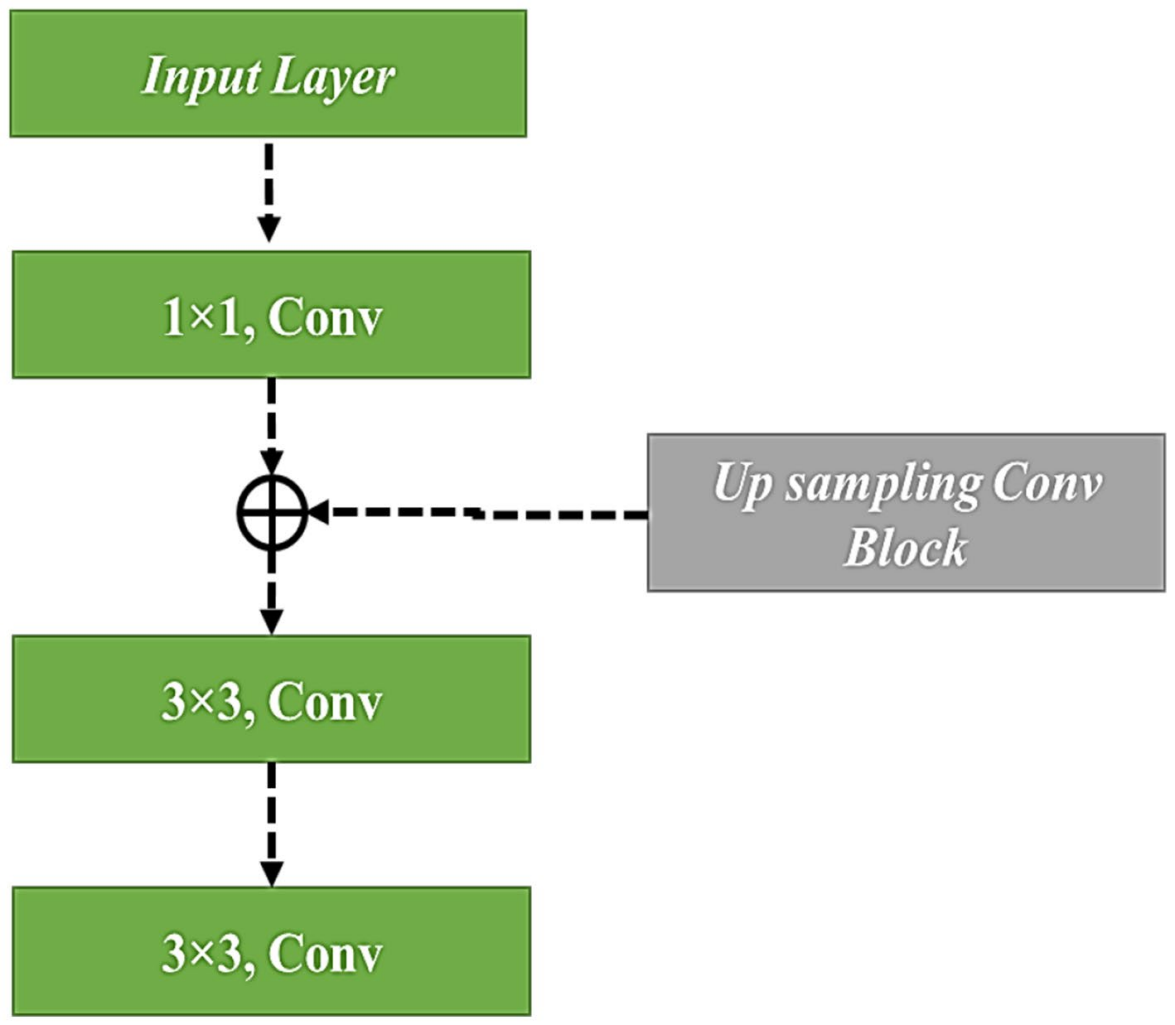
Modified bottle neck with up-sampling.

To prevent gradients from vanishing and exploding, the residual gradient structure has been used. Feature reuse is helpful for feature extraction, and residual units have been improved. During the feature extraction process, 128*128 feature information is present as the first residual block output, which has been given as the input for the 3rd residual block using downsampling, and the input scale has been changed to 75*75. Similarly, the first residual block output feature information has been sent as input, multiple downsampling has been used for the fourth residual block output, and feature size has been given as 38*38 and 19*19, respectively. The method that was used in the 1st residual block was the same as the second residual block output, which was 50*50. The subsampled output has been given to the input and output present in the fourth residual block. The residual block output is subsampled, and it has been given to the fourth residual block output. The complete representation of the modified up-sampled bottleneck process is shown in Figure 7.

In the proposed method, every block has an independent convolutional way to deliver the information present in the previous and middle layers. The strategy exhibits the concept of “pass-over,” which has been varied from ResNet, which loads the modest building blocks that contain residual connections. The classical residual building block does not use the information available in the middle layer. However, the proposed model has cached the pass-over information to obtain complete features.

The proposed model structure has been designed to achieve many features. The pass-over way leads to various feature fields, which generate features at various levels of abstraction. Moreover, it supported the ensemble effects and showed improved performance in classification.

Proposed general form of function, given in Equations 1, 2:


(1) $$g\left(x\right)=\mathrm{softmax}\left(\right)$$

(2) $$g\left(a\right)-h\left(a\right)=\left\{\begin{array}{c} a.\frac{\ln\left(e^{a}+1\right)}{1+\ln\left(e^{a}+1\right)},a\varepsilon \left(-\infty ,0\right);\\ k_{m+1}a+b_{m+1},a\varepsilon [0,a_{m+1};\\ .\\ .\\ .\\ k_{n}a+b_{n},a\varepsilon \left[a_{n}+\infty \right)\text{.} \end{array}\right.$$

During the training process of CNN, it was observed that the piecewise point of activation function was set between values of 0 and 1, greatly influencing the backward propagation of gradient, forward propagation of feature, and curve change. At point 0, the function has differentiated, and the slope of the function has been changed to 1 immediately. After conducting many tests, the piecewise function has been set as 0.1, and the function is given below in Equation 3:


(3) $$\left\{\begin{array}{c} a.\frac{\ln\left(e^{a}+1\right)}{1+\ln\left(e^{a}+1\right)}\;a\varepsilon \left(-\infty ,0\right);\\ a.\frac{\ln2}{1+\ln2}\;a\varepsilon \left[0,0.1\right);\\ a+\frac{0.1\ln2}{1+\ln2}-0.1,a\varepsilon \left[0.1,+\infty \right)\text{;} \end{array}\right.$$

At the initial stage of the test, the model exhibited overfitting directly. It was observed that the slope of the function altered quickly, and the transition of the curve’s slope from $\ln2/\left(1+\ln2\right)$ to 1 could not occur directly.

To address this, a linear function was introduced at the range (0.1, 1), acting as a buffer to stabilize the slope changes. After extensive testing, the optimal range was refined to (0.1, 0.2), which effectively mitigated the overfitting issue while preserving the model’s performance.

The modified function is as follows in Equation 4:


(4) $$\left\{\begin{array}{c} a.\frac{\ln\left(e^{a}+1\right)}{1+\ln\left(e^{a}+1\right)}\;a\varepsilon \left(-\infty ,0\right);\\ a.\frac{\ln2}{1+\ln2}\;a\varepsilon \left[0,0.1\right);\\ \left(2-\frac{\ln2}{1+\ln2}\right)x+\frac{0.2\ln2}{1+\ln2}-0.2,a\varepsilon \left[0.1,+0.2\right);\\ a,a\varepsilon \left[0.2,+\infty \right)\text{.} \end{array}\right.$$

The mean value outcome of ReLU has been compared with a new function, and the probability model of the parameter has been set as $p\left(a,\alpha \right)$ , $a^{+}$ refers to the positive input, $a^{-}$ refers to the negative input, α refers to the probability of input a . The new function output mean value after non-linear transformation is given as follows in Equations 5–8:


(5) $$E_{\mathrm{ours}}\left(a\right)=\sum \beta f_{\mathrm{ours}}\left(a\right)=\sum \beta a^{+}+\sum \beta a^{-}\text{,}$$

where


(6) $$\sum \beta a^{+}=\sum \beta a^{+}+\sum \frac{\beta ln2}{\left(1+\ln2\right)a^{+}}+\sum \beta \left[\begin{array}{l} \left(2-\frac{\ln2}{1+\ln2}\right)a^{+}+\\ \frac{0.2\ln2}{1+\ln2}-0.2 \end{array}\right]$$

(7) $$\sum \beta a^{-}=\sum \beta a^{-}.\ln\left(\exp\left[a^{-}\right]+1\right)/\left(1+\ln\left(\exp\left[a^{-}\right]+1\right)\right)$$

The output mean value of ReLU is


(8) $$E_{\mathrm{ReLU}}\left(a\right)=\sum \beta f_{\mathrm{ReLU}}\left(a\right)=\sum wa^{+}+0$$

where, $E_{\mathrm{ReLU}}\left(a\right)$ always has a positive value and the result of the new function $E_{\mathrm{ours}}\left(a\right)$ has both +ve and –ve values that make the mean value close to 0. It has accelerated the convergence of the model and updated parameters.

**Figure 8:**
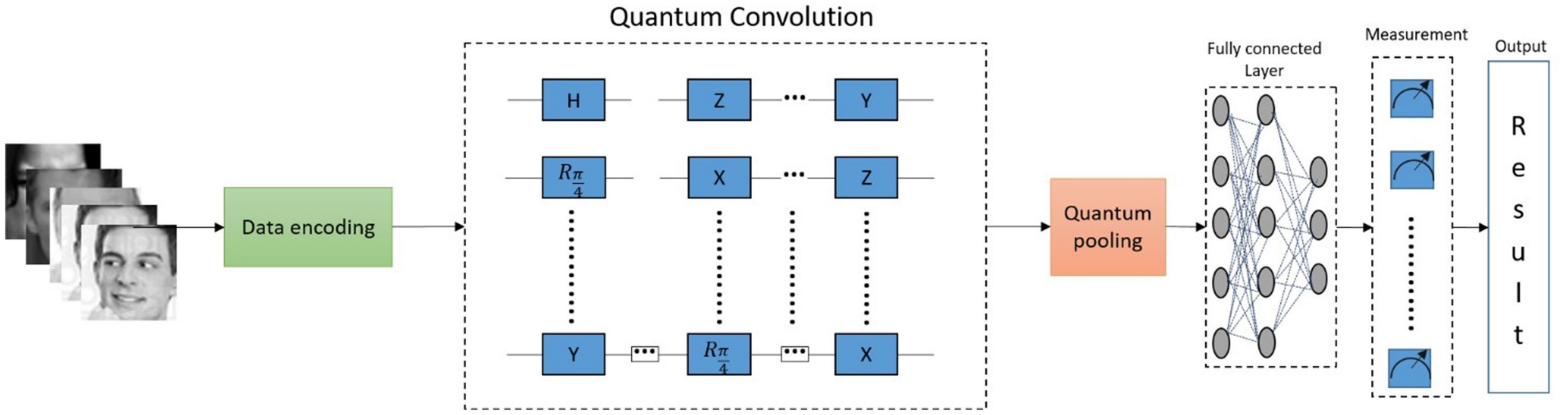
Framework of the proposed model.

Figure 8 illustrates the workflow of the proposed QCNN, where QNNs utilize quantum convolution layers and activation layers to extract features from the input images. The process begins with data encoding, converting actual images into the required quantum state. Quantum convolution is achieved by applying a series of quantum gates to the encoded state. The process continues through quantum pooling and fully connected layers, where neurons are interconnected in a feed-forward configuration, linking preceding neurons with subsequent ones. The model’s performance is evaluated, and the final quantum state is delivered as the output result.

However, integrating conventional CNN with the QCNN framework creates a hybrid model that capitalizes on the strengths of both technologies. This approach diverges from usual QCNN formats, venturing into new areas of neural network configurations as an experimental model. Furthermore, utilizing a quantum simulator to run the model and generate results represents significant progress in the practical applications of QML. The findings from the proposed study indicate that employing a quantum strategy yields superior outcomes compared to traditional techniques, as demonstrated by improved precision rates when examining face images. These findings contribute to the growing knowledge of QML, opening the door to further research and experimentation, including the application of quantum methods to tackle more complex tasks.

## 4 Results and discussion

The results that have been obtained by implementing the proposed system are included in this section, along with a dataset description, performance metrics, experimental results, performance analysis, and comparative analysis.

### 4.1 Dataset description

The study used the FER-2013 dataset, which consists of greyscale images, each with dimensions of 48*48 pixels. The images are automatically registered, meaning the faces are generally centered, and each image occupies a consistent volume of space. The goal of the study was to classify the emotions displayed in the facial expressions into one of seven categories: Neutral, Surprise, Sad, Happy, Fear, Disgust, and Angry. The dataset includes approximately 28,709 examples in the training set and 3,589 examples in the public test set. The dataset was sourced from https://www.kaggle.com/datasets/msambare/fer2013.

**Figure 9:**
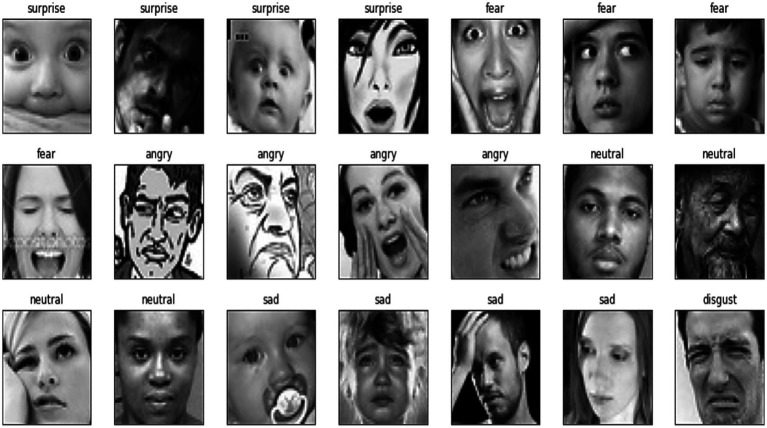
Sample images from the dataset.

The total images that are considered in the FER-2013 dataset are tabulated in Table 1 with sample images as shown in Figure 9.

**Table 1:** Total images in the FER-13 dataset.

FER2013	Total number of images
Anger	4,953
Happy	8,989
Disgust	547
Surprise	4,012
Neutral	6,198
Sad	6,077
Fear	5,121

### 4.2 Performance metrics

Performance metrics are generally used to determine the performance of the proposed model, which is calculated based on the accuracy, precision, recall, and f1-score. Performance metrics are also used to determine the proposed model’s efficiency.

- Accuracy

The term accuracy can be referred to as the model classification rate that is provided through the proportion of correctly classified instances $\left({Tru}_{Po}+{Tru}_{Ne}\right)$ to the sum of instances in the dataset $\left({Tru}_{Po}+{Fal}_{Po}+{Tru}_{Ne}+{Fal}_{Ne}\right)$ . The succeeding equation can be used to estimate the accuracy range as given in Equation 9:


(9) $$\mathrm{Accuracy}=\frac{{Tru}_{Ne}+{Tru}_{Po}}{{Tru}_{Ne}+{Tru}_{P}o+{Fal}_{Ne}+{Fal}_{Po}}$$

- Precision

The term precision is defined as the degree of covariance of the system, which results from the correctly identified instances $Tru_{Po}$ to the total number of instances that are correctly classified $\left(Tru_{Po}+Fal_{po}\right)$ . It is measured by Equation 10:


(10) $$\mathrm{Precision}=\frac{{Tru}_{Po}}{{Tru}_{Po}+{Fal}_{Po}}$$

In this equation, the variables are defined as $F\;{\mathrm{al}}_{Ne}$ -False Negative, ${Fal}_{Po}$ -False Positive, ${Tru}_{Ne}$ -True Negative, $\mathrm{and}\;{Tru}_{Po}$ -True Positive.

- F-Measure

F1-score denotes the weighted harmonic mean value of (Rec) recall and (Prec) precision. It is calculated with the following Equation 11:


(11) $$F-\mathrm{measure}=\frac{2*Rec*\mathrm{Prec}}{Rec+\mathrm{Prec}}$$

- Recall

The term recall quantifies the amount of correct positive classifications made out of all the positive classifications that are done. It is computed with the following Equation 12:


(12) $$\left(Rec\right)\mathrm{Recall}=\frac{{Tru}_{po}}{{Fal}_{Ne}+{Tru}_{Po}}$$

### 4.3 Exploratory data analysis (EDA)

In general, EDA indicates the critical procedure of performing primary investigations on the data, realizing patterns, verifying assumptions, and spotting anomalies with the help of graphical representations and summary statistics. This section deliberates on the EDA of the proposed models in the present study for the datasets FER-13. The training and test data for different emotions are mentioned in Figure 9 for better understanding.

**Figure 10:**
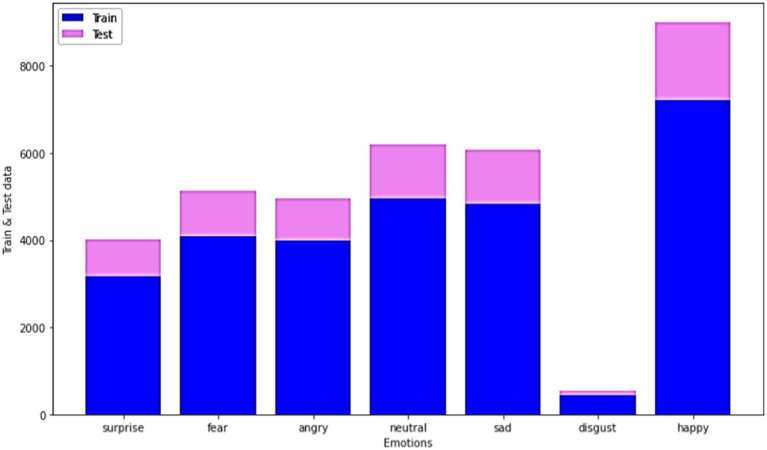
Train and test data for the FER-2013 dataset for different datasets.

For the FER-2013 dataset, sample images for some common emotions like happy, neutral, disgust, sad, angry, fear, and surprise have been shown in Figure 10. Based on the images in the dataset, the emotions are classified.

**Figure 11:**
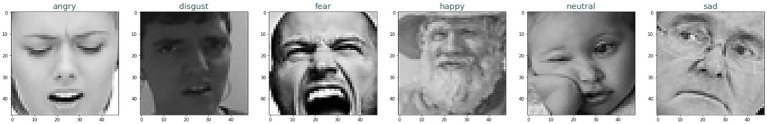
Sample images for the FER-2013 dataset with different emotions.

The test data for the FER-2013 dataset for the mentioned emotions, such as neutral, disgust, fear, anger, sadness, surprise, and happiness, has been shown in the graphical representation in Figure 11 to obtain more clarity.

**Figure 12:**
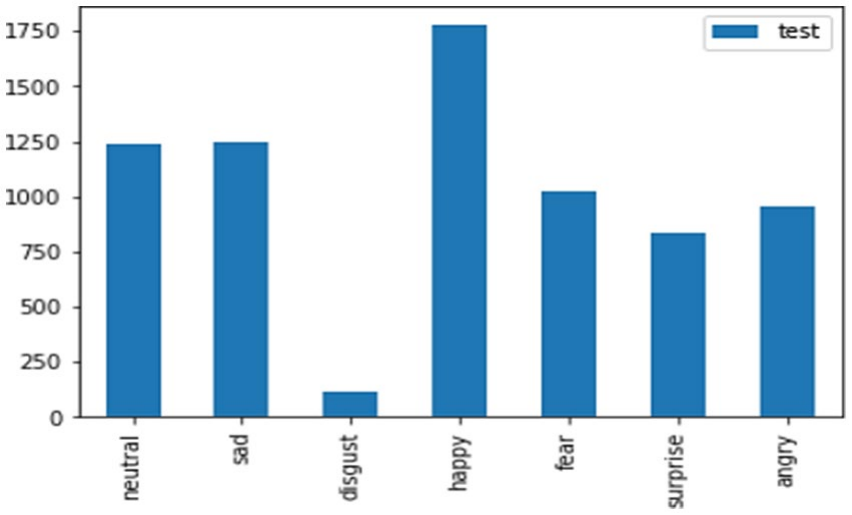
Test data for the FER-2013 dataset.

**Figure 13:**
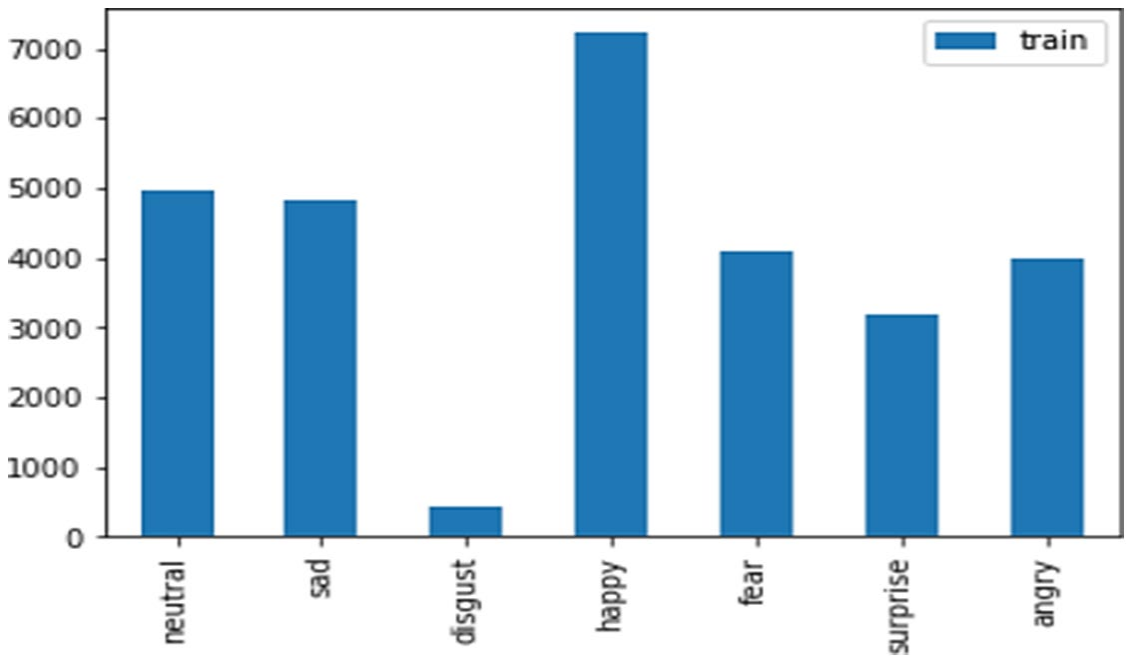
Train data for the FER-2013 dataset.

The considered train and test data for the FER-2013 dataset for the mentioned emotions like neutral, disgust, fear, anger, sad, surprise, and happy has been shown in the graphical representation in Figures 12, 13.

### 4.4 Experimental results

**Figure 14:**
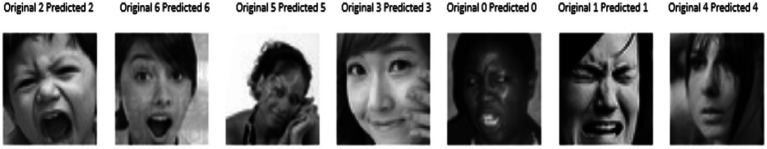
Experimental results for correct classification of the proposed model.

**Figure 15:**
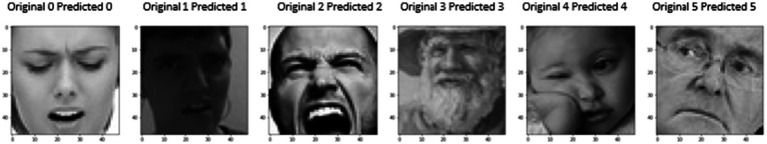
Experimental results for the correct classification of the proposed model.

The test results for the proposed model are shown in Figure 14. The proposed system, which used quantum computing and the ResNet18 architecture with modified-Up Sampled Bottle Neck Process for the FER-2013 dataset, produced the exact predictions. Figures 14, 15 clearly show that the original emotion and predicted emotions are the same. Thus, the proposed method recognizes facial emotions with utmost accuracy. The proposed method has classified the emotions into seven categories: neutral, surprised, sad, happy, fearful, disgusted, and angry. From Figure 6, it is clear that the proposed method has predicted all seven emotions correctly. On the contrary, the misclassification results are shown in Figure 15.

**Figure 16:**
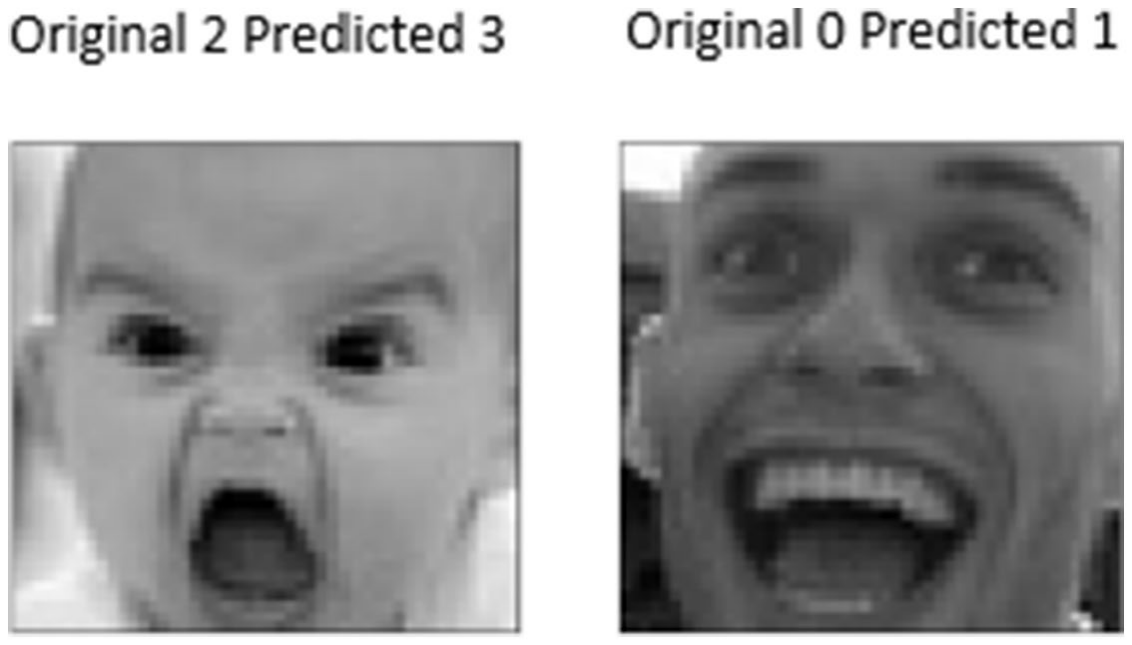
Experimental results for misclassification of the proposed model.

From Figure 16, it was found that the misclassification rate of the proposed model was 5 for the original 2.

#### 4.4.1 Statistical tests

**Figure 17:**
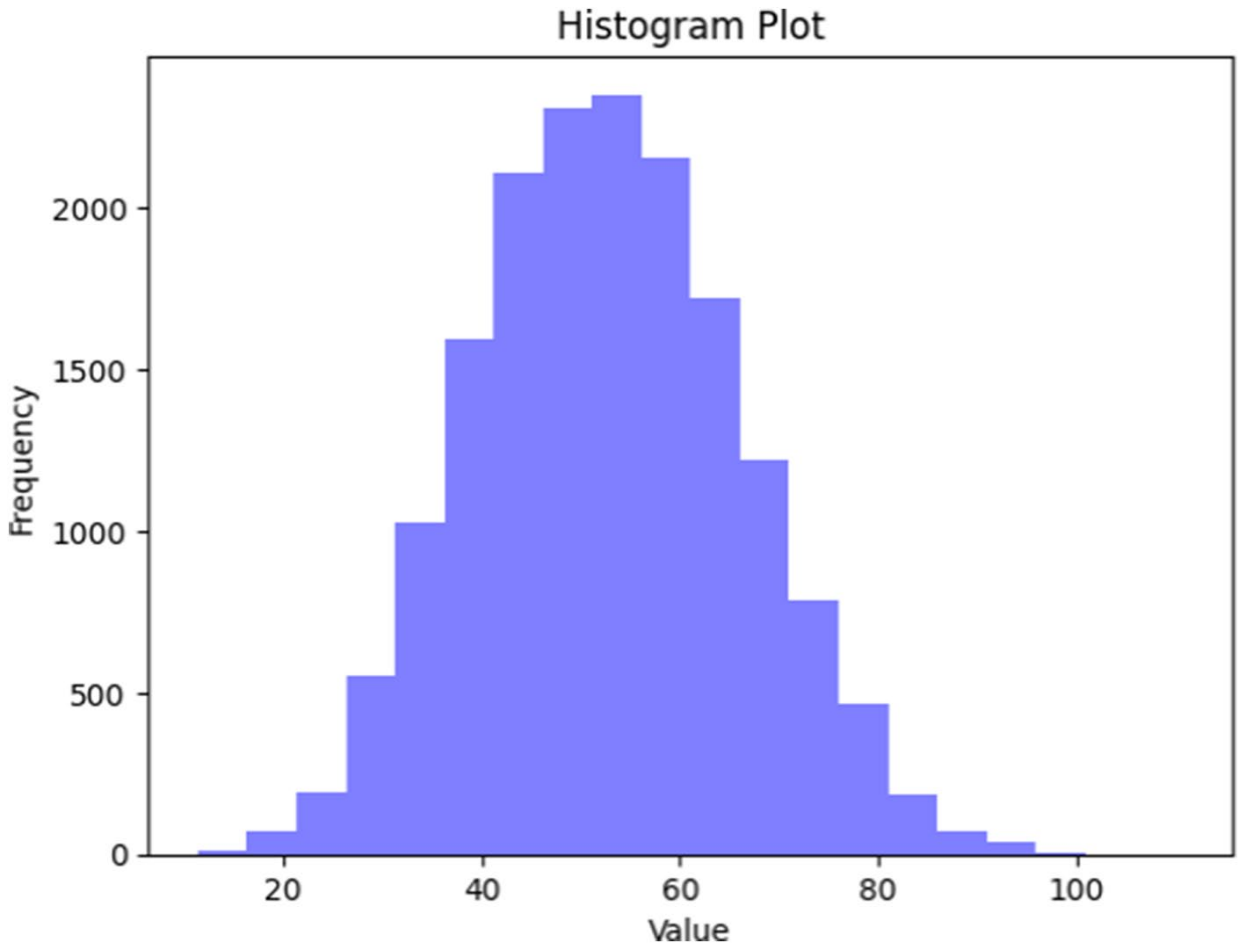
A histogram plot.

Distribution tests have been considered in this case. When the dataset pursues normal distribution, it could be found that most of the images fall within a certain SD (standard deviation) of the mean. When distribution seems to be not normal, it might be found that distribution is either skewed or possesses a heavy tail. Additionally, it is probable to evaluate if the dataset approximately pursues normal distribution with the creation of a data histogram and a visually performed inspection. Typically, a normal distribution possesses a bell-shaped curve with most of the data points clustered about the mean. When it has been assumed that FER-13 is a persistent variable (for instance, facial expression intensity), then a data histogram could be created and visually inspected for normality. When the histogram roughly pursues a bell-shaped curve, this could recommend that the dataset pursue a normal distribution. The corresponding histogram plot is shown in Figure 17.

In addition, the Shapiro–Wilk test was undertaken, which is a statistical test utilized to determine if sample data is typically distributed or not distributed. Moreover, the proposed work has used the FER 2013 BENCHMARK dataset, and the results for Shapiro–Wilk test statistics corresponding to the proposed work give 0.9844 with a *p*-value equal to 0, and it is clearly found that pixel values are not normally distributed.

### 4.5 Performance analysis

The performance of the proposed system has been analyzed, and the corresponding outcomes are discussed in this section.

**Figure 18:**
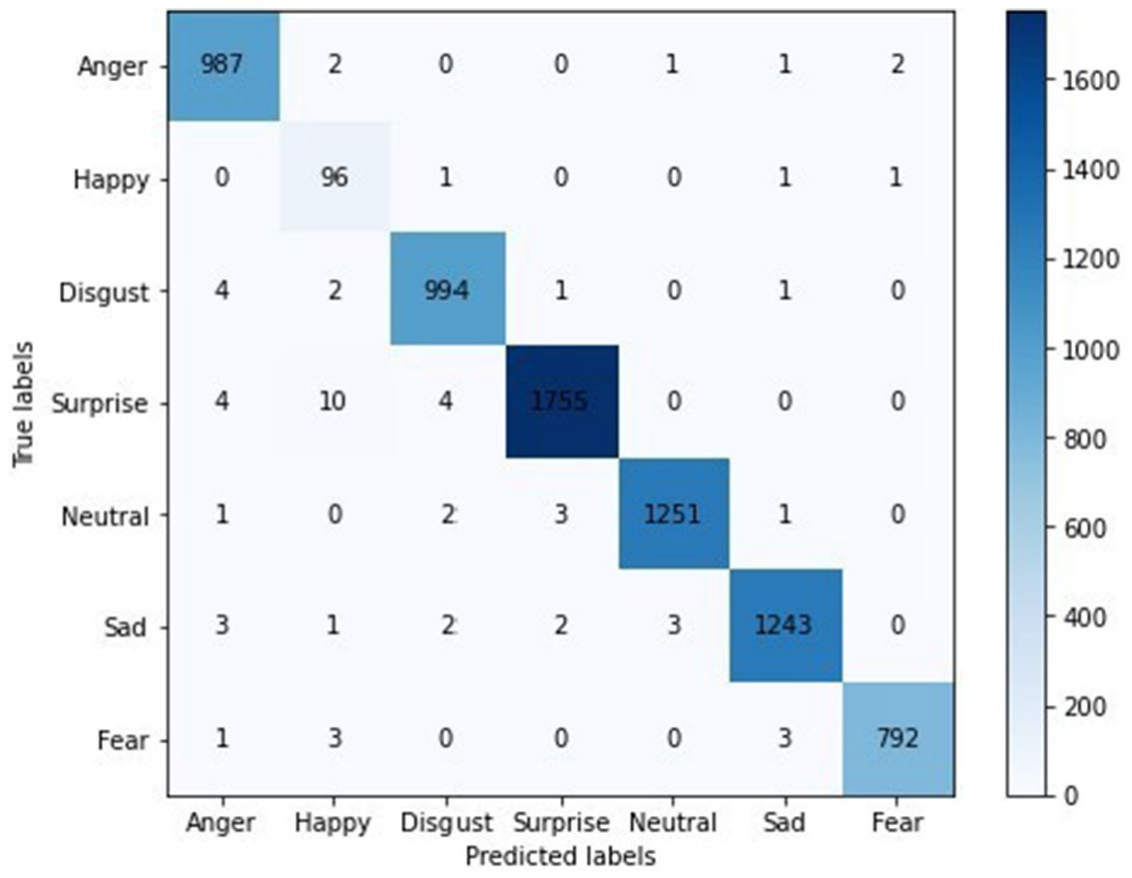
A confusion matrix.

**Figure 19:**
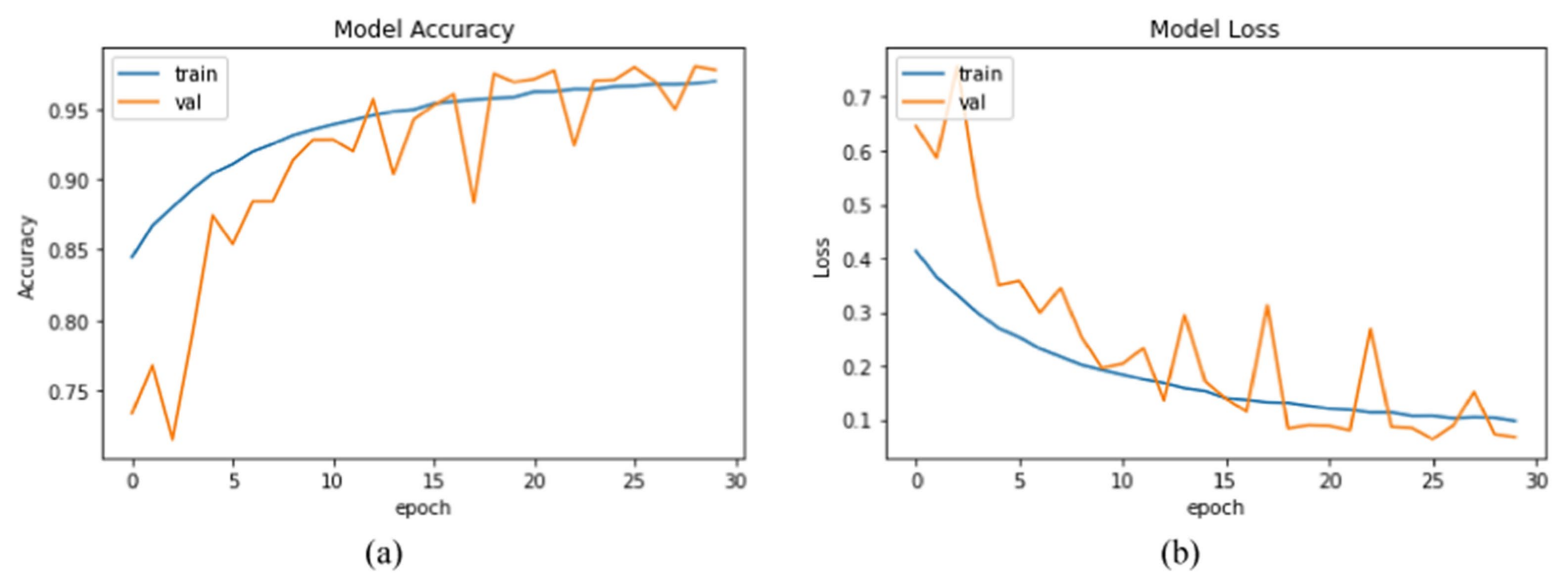
Training performance metrics of the two datasets: **(a)** accuracy analysis of the FER-2013 dataset and **(b)** loss analysis of the FER-2013 dataset.

Figure 18 shows the confusion matrix for the proposed model, illustrating the accuracy of emotion predictions. The model has successfully predicted the true labels, with “surprise” being the most accurately predicted emotion (1755 instances). In contrast, the predictions for other emotions were as follows: “neutral” (1251), “sad” (1243), “disgust” (994), “anger” (967), “fear” (762), and “happy” (96), with “happy” being the least predicted emotion. This analysis reveals that “surprise” was the most frequently and accurately identified emotion, while “happiness” had the fewest correct predictions. Moreover, Figure 19 represents the accuracy analysis of FER-2013 and shows both trained and validated accuracy.

**Figure 20:**
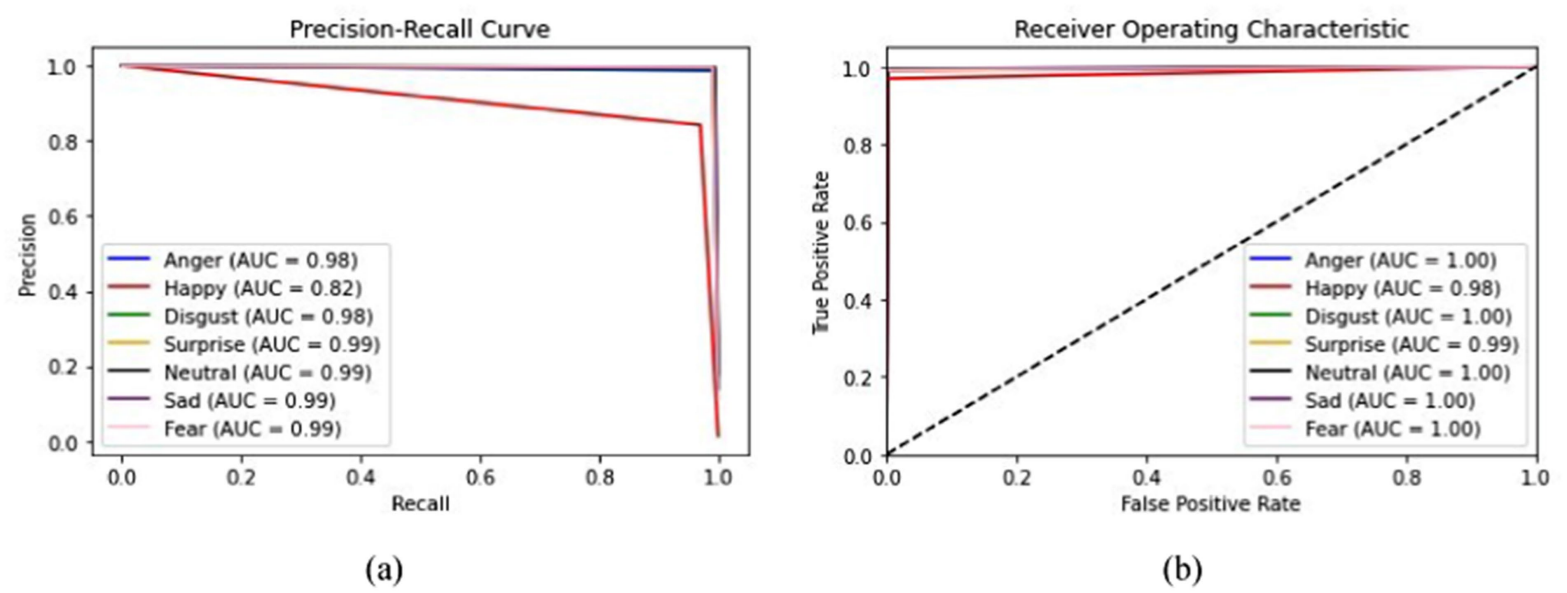
Curves visualization of the proposed model on the FER-2013 dataset **(a)** Precision-Recall curve, and **(b)** receiver operating characteristics (ROC) curve.

From Figure 19a, it is clearly visible that both train and validated accuracy have some differences until epoch 10. Train and validated accuracy have a closer match on 20, 25, and 30 epochs. Moreover, from Figure 19b, it is clearly found that both train and validated loss have some differences in epoch 0 and epoch 5. In 10,15,20,25, and 30 epochs, both train loss and validated loss have a closer match. Figure 20 visualizes the performance curves of precision-recall and receiver operating characteristics (ROC) of the proposed model on the FER-2013 dataset.

Figure 20a shows that the proposed model achieved an AUC value of 0.99 for the Precision-Recall curve for surprise, neutral, sad, and fear; 0.98 for disgust and anger; and 0.82 for happiness. The AUC curve confirms that surprise, neutral, sad, and fear have achieved high values, whereas happiness had lower prediction accuracy for the FER-2013 dataset.

Figure 20b shows that the ROC curve reached a value of 1.00 for anger, disgust, neutral, sad, and fear; 0.99 for surprise and happiness. Moreover, the performance metrics of the proposed model are tabulated in Table 2.

**Table 2:** Performance metrics of the proposed model.

Class	Precision	Recall	F1-score
Anger	0.99	0.99	0.99
Happy	0.97	0.84	0.9
Disgust	0.99	0.99	0.99
Surprise	0.99	1	0.99
Neutral	0.99	1	1
Sad	0.99	0.99	0.99
Fear	0.99	1	0.99
Accuracy			0.99
Marco Avg	0.99	0.97	0.98
Weighted Avg	0.99	0.99	0.99

For instance, the proposed model demonstrates strong performance in detecting emotions such as anger, disgust, surprise, neutral, sad, and fear, achieving precision, recall, and F1 scores close to 0.99 for each, indicating high accuracy and consistency in predicting these emotions. However, for the “happy” class, the model exhibits a distinction with a precision accuracy of 0.97 but a reduced recall rate of 0.84, leading to a slightly lower F1-Score of 0.90.

Moreover, it shows that while the model is generally accurate in predicting happiness, it fails to account for a significant number of actual happy instances. These metrics highlight the model’s strengths in most emotional categories but highlight the need for improvement in predicting happiness.

Additionally, the model achieved a kappa coefficient of 0.9899, an overall accuracy of 0.99, a macro average of 0.99 for precision, 0.97 for recall, and 0.98 for the F1-score. The weighted averages for precision, recall, and F1-score were all 0.99, further confirming the model’s robust performance.

Based on the performance analysis, the performance of the proposed system that has used quantum computing is found to be more efficient. In order to gauge its outstanding performance, the proposed system was compared with the conventional system, for which a comparative analysis was carried out. The results are discussed in the succeeding section.

### 4.6 Comparative analysis

The proposed system has been compared with four conventional studies, and the respective results are discussed in this section. The existing study has used various models such as DCNN Model1, DCNN Model2, EmNet (average fusion), and EmNet (weighted maximum fusion), and their corresponding outcomes are given in Table 3.

**Table 3:** Comparative analysis of accuracy (Saurav et al., 2021).

Model	Accuracy (%)
DCNN Model 1	72
DCNN Model 2	72.02
EmNet (average fusion)	74.11
EmNet (weighted maximum fusion)	74.06
**Proposed**	**98.19**

When compared with the existing study, we can observe that the proposed model has attained a higher accuracy of 98.19%, which is clearly shown in Table 3. The existing study (Zahara et al., 2020) has been compared with the proposed model, which used quantum computing, and the outcomes are 65.97% accuracy for the existing model and 98.19% for the proposed model. Hence, it is clear that the proposed model has better accuracy, as shown in Table 3.

The train and test accuracy of the proposed method has been compared with the existing study (Bodavarapu and Srinivas, 2021), which has used various models like FERConvNet_Gaussian, FERConvNet_Nonlocal Means, FERConvNet_Bilateral, and FERConvNet_HDM, and the outcomes are shown in Table 4.

**Table 4:** Comparative analysis of train and test accuracy (Bodavarapu and Srinivas, 2021).

Model	Train accuracy (%)	Test accuracy (%)
FERConvNet_Gaussian	98	58
FERConvNet_Bilateral	98	63
FERConvNet_Nonlocal Means	93	61
FERConvNet_HDM	98	95
**Proposed**	**99**	**98**

From Table 4, it is clear that the proposed method has attained higher train accuracy at 99%, and the test accuracy value is given as 98%, compared with the existing methods used in the existing study.

The performance metrics of the proposed method, which used quantum computing, have been compared with the existing study (Kim et al., 2021), which has used the SGD and Adam models, and it is shown that the proposed model achieves 98.19% of accuracy, 98% of precision, recall, and f1_score, compared with 76.17 and 77.17% of accuracy, 63.0118 and 66.6236% of precision, 61.0729 and 66.8845% of recall, as well as 61.0932 and 66.6779% of f1_score, respectively, for the SGD and Adam optimizers. Hence, it is clearly found that the proposed method has higher values in all performance metrics. Furthermore, a comparison has been undertaken between proposed and conventional methods by considering the JAFFE dataset. The respective outcomes are shown in Table 5.

**Table 5:** Analysis in accordance with an accuracy rate (Akhand et al., 2021).

Pre-trained deep CNN model	Accuracy (%)
VGG-16	97.62
VGG-19	98.41
ResNet-18	98.09
ResNet-34	98.57
ResNet-50	99.05
ResNet-152	99.52
Inception-v3	99.05
DenseNet-161	99.52
**Proposed model**	**99.68**

From Table 5, it can be observed that existing algorithms such as VGG-16 have revealed an accuracy rate of 97.62%, DenseNet-161 has exposed an accuracy of 99.52%, and the Inception-v3 algorithm has shown 99.05% accuracy. However, the proposed model has explored a high accuracy rate of 99.68%. Similarly, the proposed system has been compared with conventional models for the CK+ dataset (Shanthi and Nickolas, 2021), and the corresponding outcomes are 97.86% for the existing model and 98.19% for the proposed model. Hence, it can be concluded that the proposed model has been confirmed to be more effective than conventional models when considering challenging datasets like the CK+ dataset and the JAFFE dataset. Hence, from the experimental results, performance analysis, and comparative analysis, it is clearly shown that the proposed model, which used quantum computing and ResNet18 Architecture with Modified Up Sampled Bottleneck Process, shows enhanced performance with higher accuracy due to effective feature extraction.

## 5 Discussion

The study (Bursic et al., 2020) considered two models, GRU-Cell RNN and spatio-temporal CNN. These have been initially trained upon the facial features alone. It has been found that including information associated with language articulation has enhanced the accuracy rate to approximately 12%. However, the enhancement in accuracy rate has been highly reliant on the consecutive frames that have been afforded as input. Though the accuracy rate has been satisfactory, there is scope for further enhancement. Following this, the research (Qin et al., 2020) has aimed at an issue that conventional FER has not been accurate, for which CNN and GWT (Gabor Wavelet Transform) have been integrated. Initially, histogram equalization, cropping, face positioning, and several pre-processing stages were undertaken for expression images. Subsequently, keyframes corresponding to the expression sequences have been extracted. In this case, GWT was used to procure phase features, while CNN was utilized for training purposes. Experimentation has accomplished an accuracy rate of 96.81%. Furthermore, this study (El Dahshan et al., 2020) aimed to perform FER in accordance with QPSO (Quantum Particle Swarm Optimization) and DBN (Deep Belief Network). The suggested system has encompassed four stages. Initially, pre-processing has been undertaken by cropping region of interest (ROI) to attain the preferred region, thereby eliminating non-essential parts. Furthermore, image downsampling has been adapted to reduce the new sub-image size and enhance the performance of the system. Emotion class has been found with DBN. Rather than adapting the parameters of DBN manually, QPSO has been utilized to optimize DBN parameter values automatically. The suggested method has been employed in datasets including FER-2013. With the employment of the suggested system, the accuracy rate has been found to be 68.1% for the FER-2013 dataset. Furthermore, the article (Liu et al., 2020) has encompassed three major phases: frontal face identification module, feature extraction, and classification. Feature extraction encompasses dual channels. In this case, one is for raw facial images, while the other one seems to be for the extraction of features from the images. LBP images have been utilized to extract texts to enrich the facial features, thereby improving the performance of the network. Furthermore, an attention mechanism has been adopted. Moreover, the arc-face loss function has been included for improvising the distance of the inter class and minimizing the distance of the inner class. Experimentations have been undertaken on two accessible datasets, namely CK+ and FER-2013. Outcomes have revealed an accuracy rate of 94.24% for the CK+ dataset and 72.56% for the FER-2013 dataset. In spite of various endeavors undertaken by existing works, it has been clearly found that there is a scope for enhancement with regard to accuracy. Accordingly, the proposed system has shown better results in accordance with accuracy (98.19%) than conventional systems.

### 5.1 Ethical implications of FER

Ethical concerns tied to FER technology, such as privacy, consent, and potential abuse, are significant. FER technology could enhance user interactions in various fields, such as healthcare and security, but it also poses risks like privacy invasion and the possibility of misidentification or bias, especially toward marginalized groups. To encourage ethical use, it is crucial to set up protocols such as obtaining consent before collecting emotional data, explaining the data’s purpose, and conducting regular assessments to detect and correct algorithm biases. Additionally, the establishment of regulatory frameworks can help monitor the deployment of FER technologies, ensuring their ethical application and preventing infringements on fundamental rights. By prioritizing these approaches, individuals can reap FER’s advantages, minimize its drawbacks, and establish trust with the public.

## 6 Conclusion

This study aimed to detect emotions from facial expressions using quantum computing. The experimental results showed that quantum computing performs more effectively, even with large and complex datasets. The FER-2013 dataset used in the research and ResNet18 Architecture with Modified Up-Sampled Bottleneck Process were used to classify emotion types from the provided emotions, such as neutral, disgust, anger, sad, happy, surprise, and fear. The proposed system performance was evaluated based on four performance metrics, and the outcomes were found to be 98.19% accuracy, 98% recall, 98% f1-score, and 98% precision. Furthermore, comparative analyses were undertaken with four recent studies to confirm the efficacy of the proposed system. The outcomes of the analysis showed that the proposed model had better values in the performance metrics when compared with the existing models. The results showed the efficient performance of the proposed system over the existing models, and the proposed method achieved 98.19% accuracy. Furthermore, the standard deviation of the proposed system was determined from the execution of the proposed system and was found to be 52.69816460460272. Moreover, the computational complexity for QNNs typically relies on the depth and size of the circuit, the dimensionality of input, and the number of training samples. Accordingly, for ResNet18, the computational complexity is O(n^2^.d), where *n* represents the length of image features and *d* corresponds to the quantum bit dimension. With the integration of position encoding, computational complexity increases to O (n2.d + n.d2). Future studies should further explore the power of quantum computing in machine learning applications.

## Data Availability

Publicly available datasets were analyzed in this study. This data can be found at: https://www.kaggle.com/datasets/msambare/fer2013.
